# AIM2 inflammasome regulated by the IFN‐γ/JAK2/STAT1 pathway promotes activation and pyroptosis of monocytes in Coronary Artery Disease

**DOI:** 10.1002/iid3.1317

**Published:** 2024-06-13

**Authors:** Yue Zhao, Bin Liang, Shuyang Sheng, Chen Wang, Bingyu Jin, Xiaokang Zhang, Yating Cheng, Changxin Shen, Fang Zheng

**Affiliations:** ^1^ Center for Gene Diagnosis and Department of Clinical Laboratory Medicine Zhongnan Hospital of Wuhan University Wuhan China; ^2^ Department of Clinical Laboratory the First Affiliated Hospital of Zhengzhou University Zhengzhou China; ^3^ Key Clinical Laboratory of Henan Province Zhengzhou China; ^4^ Department of Blood Transfusion Zhongnan Hospital of Wuhan University Wuhan China

**Keywords:** AIM2, Coronary Artery Disease, IFN‐γ/JAK2/STAT1 pathyway, inflammasome, monocytes, pyroptosis

## Abstract

**Background:**

Numerous studies have demonstrated that Absent in Melanoma 2 (AIM2) is upregulated in aortic plaques, especially in Vascular Smooth Muscle Cells in Coronary Artery Disease (CAD), and is related to inflammasome‐induced inflammation. However, the underlying mechanism of this phenomenon and the role of AIM2 in atherosclerosis remained unclear.

**Methods:**

This study enrolled 133 CAD patients and 123 controls. We isolated Peripheral Blood Leukocytes (PBLs) and the mRNA expression of AIM2 inflammasome and its downstream genes (ASC, Caspase‐1, IL‐1β, and IL‐18) were detected by real‐time quantitative PCR (qPCR). We assessed correlations between AIM2 expressions and clinical characteristics by multiple linear regression and spearman's correlation. The THP‐1 cells cultured in poly(dA:dT), A151, interferon‐gamma (IFN‐γ), AG490, or JC2‐11. And then the mRNA and protein levels of AIM2, ASC, Caspase‐1, IL‐1β, IL‐18, GSDMD, and STAT1 were analyzed by qPCR and Western blot analysis, respectively. The migration and adhesive capacity of THP‐1 cells was assessed using an inverted microscope and an inverted fluorescence microscope, respectively.

**Results:**

In this study, we found that expressions of components of AIM2 inflammasome and its downstream genes (ASC, Caspase‐1, IL‐1β, and IL‐18), were all increased in PBLs of CAD patients, which indicated the inflammasome activation. AIM2 inflammasome activation further induced pyroptosis, and stimulated migration and adhesion in monocyte cell lines, which was regulated by IFN‐γ probably through JAK2/STAT1 pathway. In addition, AIM2 expressions were positively correlated with systemic inflammatory indicators as an independent risk factor for CAD.

**Conclusions:**

In conclusion, increased AIM2 expression, induced by the IFN‐γ/JAK2/STAT1 signal, orientates monocytes to inflammatory status or even pyroptosis through AIM2 inflammasome activation, which is involved in the development of CAD.

## Graphical abstract

1. The expression of Absent in Melanoma 2 (AIM2) were increased in Peripheral Blood Leukocytes (PBLs) of Coronary Artery Disease (CAD) patients. 2. AIM2 expression was positively correlated with systemic inflammatory indicators as an independent risk factor for CAD. 3. AIM2 AIM2 inflammasome activation induced monocyte pyroptosis and stimulated the migration and adhesive capacity of monocyte.

## 1 INTRODUCTION

Coronary Artery Disease (CAD) has become a major cause of death in modern society.^1^ Despite advances in prevention and treatment of CAD, it remains a threat to human health worldwide mostly due to the Residual Inflammation Risk.^2^ Canakinumab Anti‐Inflammatory Thrombosis Outcome Study (CANTOS) showed that it was able to stabilize plaque well and prevent the occurrence of Cardiovascular (CV) events when blocking the inflammatory process.^3^ Monocytes are one of the most critical immunology cells in the chronic inflammatory process of atherosclerosis.^4^, ^5^ In addition, immunology cells including T cells, mast cells, dendritic cells, macrophages, and so forth. are all involved in this process, by amplifying the production and signaling of cytokines that regulate plaque formation and growth, for example, by producing large amounts of Interferon‐gamma (IFN‐γ) and Tumor Necrosis Factor‐α (TNF‐α).^6^, ^7^

IFN‐γ was found to activate Absent in Melanoma 2 (AIM2) inflammasome in human keratinocytes through the JAK2‐STAT1 pathway, causing a downstream inflammatory response that leads to inflammatory skin diseases.^8^ Whether IFN‐γ could activate AIM2 inflammasome in monocytes to induce inflammation in CAD need further investigation.

AIM2 is a member of the hematopoietic IFN inducible nuclear protein family, containing a 200‐amino acid repeat (HIN‐200). It can recognize various endogenous and exogenous Double‐Strand DNA (dsDNA) that can trigger inflammasome activation. Upon activation, AIM2 starts to assemble the inflammasome complex, consisting of pro‐caspase‐1 and apoptosis associated speck‐like protein containing a CARD (ASC). Once assembled, the AIM2 inflammasome can activate caspase‐1, promote the production of IL‐1β and IL‐18 and induce GSDMD‐mediated cell pyroptosis.^9^, ^10^ Sustained activation of AIM2 inflammasome leads to inflammatory damage of tissues.^11^ Recent in vivo studies suggest that AIM2 inflammasome plays a role in various CV events.^12^, ^13^ For example, Pan et al. found that Vascular Smooth Muscle Cells respond to inflammatory signals by upregulating the expression of AIM2.^14^

**Figure 1:**
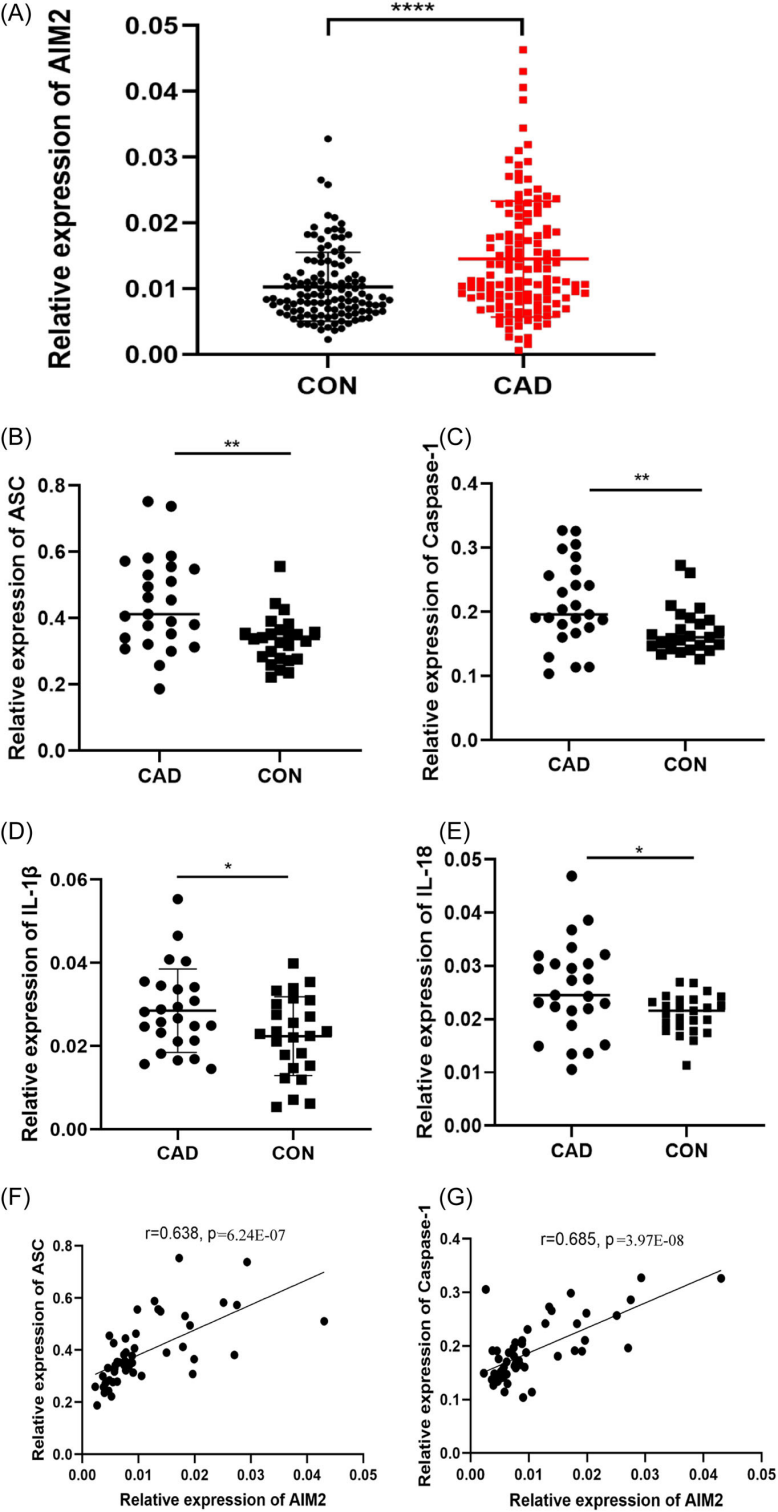
Elevated expression of AIM2 and downstream genes in PBLs of CAD patients. (A) The mRNA levels of AIM2 were detected by qPCR in the PBLs of CAD patients (*n* = 133) and controls (*n* = 123). The mRNA levels of (B) ASC, (C) Caspase‐1, (D) IL‐1β, and (E) IL‐18 were detected by qPCR in the PBLs of partial CAD patients (*n* = 25) and controls (*n* = 25). The correlations of expressions of AIM2 and ASC (F) or Caspase‐1 (G) in all subgroup participants. Significant was defined as *p* < 0.05. *p < 0.05; **p < 0.01; ****p < .0001. CAD, Coronary Artery Disease; PBLs, Peripheral Blood Leukocytes.

Recently, we found the AIM2 gene was significantly overexpressed in the CAD group in GSE42148 of Peripheral Blood Leucocytes (PBLs) in the GEO database which included 11 controls and 11 CAD patients (Supporting Information S1: Figure 1). We hypothesized that overexpressed AIM2 is probably regulated by IFN‐γ/JAK2/STAT1 pathway, and played an essential role in the inflammatory responses and activation of monocytes. So, we executed the following study to explore the function of AIM2 in monocyte inflammatory in CAD.

## 2 MATERIALS AND METHODS

### 2.1 Clinical cohorts

Two hundred fifty‐six study subjects, including 133 CAD patients and 123 controls, were recruited at Zhongnan Hospital of Wuhan University (Wuhan, China). All subjects were unrelated Chinese Han individuals, whose age and gender were matched between patients and controls. The inclusion and exclusion criteria were consistent with previous studies.^15^ The clinical information of all subjects, including age, sex, medical histories, routine blood parameters, biochemical indices, and other clinical data, was collected. And, the diagnostic criteria for hypertension, hyperlipidemia and Type 2 Diabetes (T2DM) were also followed our previous study.^16^ The present study was authorized by the Medical Ethics Committee of Zhongnan Hospital of Wuhan University (2020195) and followed the Declaration of Helsinki.

### 2.2 Reagents and antibodies

Poly(dA:dT)/LyoVecTM were obtained from Invitrogen and recombinant A151 (5’‐TTAGGGTTAGGGTTAGGGTTAGGG‐3’) constructs (AIM2 inhibitor) were synthesized with a phosphorothioate backbone unless otherwise specified at TAKARA. Human IFN‐γ was purchased from PeproTech. AG490 (JAK2 inhibitor) and JC2‐11(inflammasome inhibitor) were obtained from MedChemExpress. Poly (dA:dT)/LyovecTM and human IFN‐γ were dissolved in sterile endotoxin‐free water. A151 was dissolved in TE buffer. AG490 and JC2‐11 was dissolved in DMSO. Antibodies against AIM2, Caspase‐1, GSDMD, GAPDH, and horseradish peroxidase‐conjugated secondary antibodies were obtained from ABclonal. Antibody against p‐STAT1 (Tyr‐701) was purchased from Abcam.

### 2.3 Isolation of primary monocytes

Sorting monocyte assay was performed using a magnetic separation column (Miltenyibiotec). After collection of 7 mL of peripheral blood from volunteers, Peripheral Blood Mononuclear Cells (PBMC) were separated by standard density gradient centrifugation.^17^ Then 80 μL of magnetic bead separation buffer (Miltenyibiotec) and 20 μL of magnetic beads coated with CD14 antibody (eBioscience) were added to 1 × 10^7^ PBMC. The cells separated from the magnetic beads were obtained as monocytes, according to the instructions.

### 2.4 Cell culture

Cell lines were obtained from China Center for Type Culture Collection (CCTCC). THP‐1 cells were cultured in RPMI 1640 medium (Gibco) containing 10% Fetal Bovine Serum (FBS) (Gibco), 1% Penicillin‐Streptomycin Solution (PS) (Gibco). Human umbilical vein endothelial cell lines (HUVECs) were cultured in DMEM medium (Gibco) containing 10% FBS and 1% PS. The isolated monocytes from blood were cultured in RPMI 1640 medium containing 1% PS and 15% FBS. All cells were incubated at 37°C in a 5% CO_2_ incubator.

### 2.5 RNA extraction and real‐time quantitative PCR (qPCR)

The total RNA of cells was extracted using TRIzol reagent (Invitrogen) following the manufacturer's instructions. The RNA was reverse‐transcribed into cDNA by PrimeScriptTM RT reagent Kit with gDNA Remover (Takara). Real‐time qPCR was performed using SYBR Prime Script qPCR kit (CWBIO), specific primers on Bio‐Rad CFX 96 real‐time system (Biorad). The primer sequences are shown in Supporting Information S1: Table 1. The relative expression was calculated using the comparative crossing threshold cycle threshold Ct ($2^{-\Delta \Delta C_{t}}$) method with GADPH as the internal reference control.

### 2.6 Protein extraction and Western blot analysis

The total protein of cells was extracted using a protein lysis solution (Beyotime). Each well was spiked with 15 μg of total protein, separated by 10% SDS‐polyacrylamide gel electrophoresis (SDS‐PAGE). The protein bands were transferred to the PVDF membrane, then blocked with 5% bovine serum albumin for 2 h at room temperature, before incubation at 4°C overnight with primary antibody dilutions of AIM2, caspase‐1, GSDMD or p‐STAT1, followed by incubation with secondary antibody dilutions. The PVDF membrane was developed by ECL luminol (eBioscience). The grayscale values of the specific bands were analyzed using ImageJ software.

### 2.7 Transwell assay

The migration assay was performed using Transwell insert plates (Corning). About 200 μL of serum‐free RPMI 1640 medium containing approximately 5 × 10^5^ cells are added to the upper chamber, while 600 μL of RPMI 1640 medium containing 200 ng/mL monocyte chemoattractant protein‐1 and 5% FBS was added to the lower chamber. The chambers were incubated for 90 min at 37°C in an incubator with 5% CO_2_. The culture medium was discarded and the chambers were fixed in 800 μL of 4% paraformaldehyde (Biosharp, Beijing, China) for 10 min at room temperature, followed by the addition of crystal violet staining solution (Beyotime). Then several random fields were chosed and photographed under an inverted microscope at a magnification of ×100. The number of THP‐1 cells in each area was calculated using the ImageJ software.

### 2.8 Adhesion assay

The THP‐1 cells were incubated with the BCECF‐AM fluorescent probe (Beyotime) for 30 min in a CO_2_ incubator hidden from light, washed with PBS to remove the excess probe, resuspended in serum‐free 1640 medium, and added to fully adherent fused HUVEC in 12‐well plates at a density of 5 × 10^5^ cells/mL for 1 h. The nonadherent THP‐1 cells were washed away with PBS. THP‐1 cells were observed under an inverted fluorescence microscope at a magnification of ×100, and several randomly selected areas were photographed. The number of THP‐1 cells in each area was calculated using the ImageJ software.

### 2.9 Statistical analysis

Kruskal−Wallis test was used to analyze the normal distribution. Continuous variables that obeyed normal distribution were expressed as mean ± standard deviation, continuous variables that did not obey normal distribution were expressed as median (interquartile spacing), and categorical data were expressed as frequencies (percentages). Continuous variables were tested with Student's *t*‐test or Mann−Whitney *U*‐test, and categorical variables were tested with a chi‐square test. Multiple linear regression, as well as Spearman's correlation, were used to analyze correlations between AIM2 and clinical data. Univariate and binary logistic regression were used to analyze associations between AIM2 and CAD. All analyses were performed with SPSS 22.0 software and GraphPad Prism 8.0. A *p* < 0.05 was considered statistically significant.

## 3 RESULTS

### 3.1 Clinical characteristics

The basic characteristics of all participants are shown in Table 1. CAD patients and controls were matched for age and sex (*p* = 0.063, 0.392). CAD patients had a higher prevalence of hypertension, hyperlipidemia and T2DM than controls (*p* = 9.40E‐09, 2.17E‐20, 0.001). Also, TG and FPG were higher but HDL‐C levels were lower in patients than controls (*p* < 0.0001). Compared with controls, the percentage of neutrophils and monocytes was significantly increased in CAD patients, while the rate of lymphocytes was significantly decreased.

**Table 1:** Clinical characteristics of the CAD and control (non‐CAD) individuals.

Clinical data	CAD (*n* = 133)	Control (*n* = 123)	*p*
Male, *n* (%)	89 (66.92)	76 (61.79)	0.392^a^
Age, years	62 (55.67)	58 (52.65)	0.063^b^
Hypertension, *n* (%)	82 (61.65)	37 (30.08)	9.40E‐09^a^
Hyperlipidemia, *n* (%)	68 (51.13)	0 (0)	2.17E‐20^a^
T2DM, *n* (%)	28 (21.05)	8 (6.50)	0.001^a^
FPG, mmol/L	5.80 (5.22, 6.73)	5.50 (5.13, 5.78)	<0.0001^c^
TC, mmol/L	4.16 (3.46, 5.30)	4.49 (4.02, 4.85)	0.297^c^
TG, mmol/L	1.31 (0.97, 1.83)	1.10 ± 0.32	<0.0001^c^
HDL‐C, mmol/L	1.09 (0.87, 1.30)	1.35 (1.21, 1.59)	<0.0001^c^
LDL‐C, mmol/L	2.62 ± 0.91	2.68 (2.26, 3.04)	0.273^c^
WBC, 10^9^/L	5.80 (5.22, 6.73)	5.64 (4.80, 6.57)	0.388^c^
NEU, 10^9^/L	62.97 (55.78, 69.08)	54.67 ± 7.36	<0.0001^c^
LYM, 10^9^/L	25.46 (20.00, 31.66)	35.18 ± 6.84	<0.0001^c^
MON, 10^9^/L	7.97 (6.68, 9.09)	7.05 ± 1.30	<0.0001^c^
PLT, 10^9^/L	197.39 ± 55.48	205.33 ± 43.29	0.157^c^

*Note*: Significant was defined as *p* < .05. Data were presented as mean ± standard deviation, median (25 percentiles, 75 percentiles).

Abbreviations: FPG, fasting plasma glucose; HDL‐C, high‐density lipoprotein cholesterol; LDL‐C, low‐density lipoprotein cholesterol; LYM, lymphocyte; MON, monocyte; NEU, neutrophil; PLT, platelet; TC, total cholesterol; TG, triglyceride; T2DM, type 2 diabetes; WBC, white blood cell.

^a^ *χ* ^2^ test.

^b^ Two‐tailed Student's *t* test.

^c^ Nonparametric test (Mann−Whitney *U* test).

We also randomly selected 25−25 age‐sex matched subgroups among them for further investigation on the downstream signaling pathway. The clinical characteristics of these individuals in subgroups are shown in Table 2.

**Table 2:** Clinical characteristics of the CAD and control (non‐CAD) individuals involved in Sub‐group analysis.

Clinical data	CAD (*n* = 25)	Control (*n* = 25)	*p*
Male, *n* (%)	18 (72)	15 (60)	0.392^a^
Age, years	59.96 ± 9.7	55.88 ± 9.46	0.139^b^
Hypertension, *n* (%)	17 (68)	9 (36)	0.012^a^
Hyperlipidemia, *n* (%)	14 (56)	0 (0)	1.00E‐05^a^
T2DM, *n* (%)	4 (16)	1 (4)	0.157^a^
FPG, mmol/L	5.74 (5.33, 7.26)	5.50 (5.01, 5.79)	0.057^c^
TC, mmol/L	4.52 ± 1.57	4.17 ± 0.72	0.329^b^
TG, mmol/L	1.54 (0.88, 2.80)	1.18 ± 0.38	0.087^c^
HDL‐C, mmol/L	1.28 ± 0.50	1.35 ± 0.30	0.548^b^
LDL‐C, mmol/L	2.79 ± 1.16	2.66 (2.38, 3.02)	0.98^c^
WBC,10^9^/L	6.31 ± 1.85	5.96 ± 1.41	0.461^b^
NEU,10^9^/L	61.09 ± 8.62	55.33 ± 8.53	0.023^b^
LYM,10^9^/L	27.39 ± 7.92	34.23 ± 7.73	0.004^b^
MON,10^9^/L	8.52 ± 1.65	7.15 (6.43, 8.30)	0.005^c^
PLT,10^9^/L	195.87 ± 41.52	199.67 ± 44.00	0.757^b^

*Note*: Data were presented as mean ± standard deviation, median (25 percentiles and 75 percentiles).

Abbreviations: FPG, Fasting Plasma Glucose; HDL‐C, High‐Density Lipoprotein Cholesterol; LDL‐C, Low‐Density Lipoprotein Cholesterol; LYM, Lymphocyte; MON, Monocyte; NEU, Neutrophil; PLT, Platelet; T2DM, Type 2 Diabetes; TC, Total Cholesterol; TG, Triglyceride; WBC, White Blood Cell.

^a^ *χ* ^2^ test. Significant was defined as *p* < 0.05.

^b^ Two‐tailed Student's *t* test.

^c^ Nonparametric test (Mann−Whitney *U* test).

### 3.2 Elevated expressions of AIM2 and its downstream genes in PBLs of CAD patients

The expression of AIM2 in CAD patients was significantly increased compared to controls (133 vs. 123, *p* = 4.2653E‐7) (Figure 1A). The expression of downstream genes including ASC, Caspase‐1, IL‐1β and IL‐18 in CAD was also significantly increased in the CAD subgroup compared with the control subgroup (25 vs. 25, *p* = 0.001, .005, .02, .01) (Figure 1B−E). Moreover, expressions of ASC and Caspase‐1 were positively correlated with the AIM2 expression (*r*_ASC_ = 0.638, *p* = 6.24E‐07; *r*_Caspase‐1_ = 0.685, *p* = 3.97E‐08) in the combined patient and control subgroup (*n* = 50) (Figure 1F,G). There was no significant correlation between AIM2 and IL‐1β or IL‐18 (The results were not shown).

### 3.3 Correlations between AIM2 expressions and clinical characteristics

**Figure 2:**
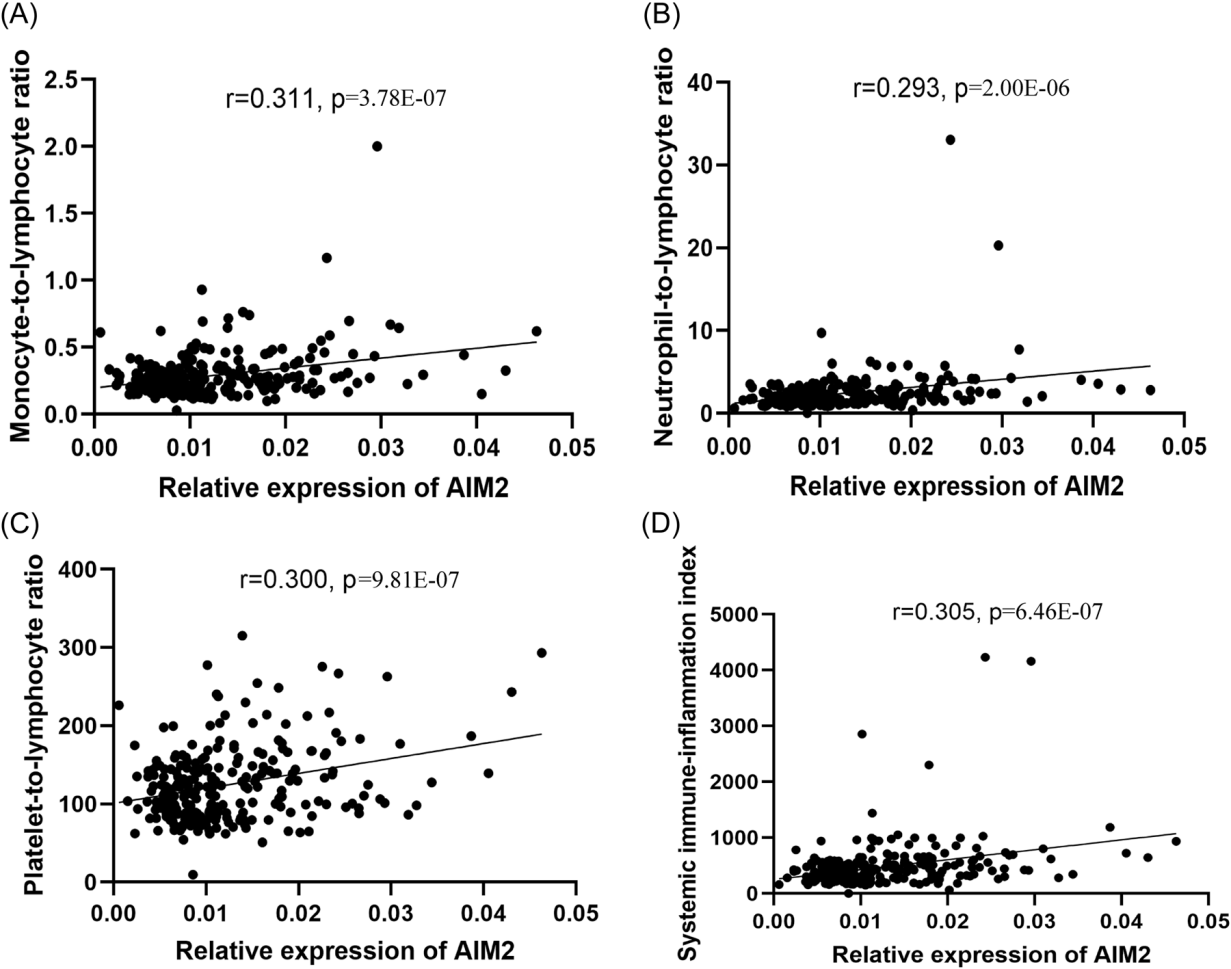
The correlations of mRNA level of AIM2 and MLR (A), NLR (B), PLR (C), and SII (D) in all participants. AIM2, Absent in Melanoma 2; CAD, Coronary Artery Disease; MLR, Monocyte‐to‐Lymphocyte Ratio; mRNA, messenger RNA; NLR, Neutrophil‐to‐Lymphocyte Ratio; PLR, Platelet‐to‐Lymphocyte Ratio; SII, Systemic Immune‐Inflammation Index.

To further investigate the clinical significance of AIM2, we assessed correlations between AIM2 expressions and the clinical characteristics of all participants. As displayed in Table 3, AIM2 was positively correlated with age (*r* = 0.292, *p* = 1.98E‐06), FPG (*r* = 0.150, *p* = 0.019), WBC (*r* = 0.128, *p* = 0.041), NEU (*r* = 0.348, *p* = 1.10E‐08), but negatively correlated with LYM (*r* = −0.334, *p* = 4.50E‐08) in all participants. After the interferences of age, FPG, WBC and LYM were adjusted by multivariate stepwise linear regression, AIM2 was still associated with NEU (*β* = 0.343, *p* = 3.50E‐08). Further, we analyzed correlations between AIM2 and systemic inflammatory indicators in all participants, including Monocyte‐to‐Lymphocyte Ratio (MLR), Neutrophil‐to‐Lymphocyte Ratio (NLR), Platelet‐to‐Lymphocyte Ratio (PLR) and Systemic Immune‐Inflammation Index (SII). It was found that AIM2 expression was positively correlated with MLR (*r* = 0.311, *p* = 3.78E‐07), NLR (*r* = 0.293, *p* = 2.00E‐06), PLR (*r* = 0.300, *p* = 9.81E‐07) and SII (*r* = 0.305, *p* = 6.46E‐07) (Figure 2).

**Table 3:** Correlation analysis and multivariate stepwise linear regression analysis of the AIM2 expression with clinical parameters of all participants.

Clinical characteristics	AIM2 expression			
	Spearman correlation		Multiple linear regression	
	*r*	*p*	*β*	*p*
Male	−	−	−	−
Age	0.292	1.98E‐06	−	−
Hypertension	−	−	−	−
Hyperlipidemia	−	−	−	−
T2DM	−	−	−	−
FPG	0.150	0.019		
WBC	0.128	0.041		
NEU	0.348	1.10E‐08	0.343	3.50E‐08
LYM	−0.334	4.50E‐08	−	−
MON			−	−
PLT	−	−	−	−

*Note*: Significant was defined as *p* < 0.05.

### 3.4 High expression of AIM2 is an independent risk factor for CAD

To investigate the association between the AIM2 expression and CAD, we divided 256 subjects to the high and low AIM2 expression subgroups according to the median of AIM2 expressions and performed a logistic regression analysis of AIM2 expressions and the prevalence of CAD. The results showed that the Odds Ratio (OR) of CAD for high AIM2 expressions (OR = 2.519, 95% Confidence Interv (CI) = 1.522‐4.168, *p* = 3.24E‐04) were significant (Table 4). Moreover, AIM2 expressions were still an independent risk factor (OR = 4.564, 95% CI, 2.115 to 9.850, *p* = 1.10E‐04) of CAD after adjusting age, sex, T2DM, hyperlipidemia and hypertension.

**Table 4:** Relationships between AIM2 mRNA expression and CAD.

Variable	AIM2		
	High‐expression	Low‐expression	*p*
Not adjusted	2.519 (1.522, 4.168)	1	3.24E‐04
Adjusted for age, sex	2.575 (1.511, 4.386)	1	5.02E‐04
Plus histories of T2DM	2.818 (1.615, 4.915)	1	2.63E‐04
Plus histories of Hyperlipidemia	4.224 (2.038, 8.753)	1	1.06E‐04
Plus histories of Hypertension	4.564 (2.115, 9.850)	1	1.10E‐04

*Note*: The results are presented as OR (95% CI); The patients with low expression of AIM2 were as controls. Significant was defined as *p* < .05.

Abbreviations: AIM2, Absent in Melanoma 2; CAD, Coronary Artery Disease; CI, Confidence Interval; mRNA, messenger RNA; OR, Odds Ratio.

### 3.5 AIM2 inflammasome promotes inflammatory responses and causes pyroptosis in human monocyte cell lines

To reveal the biological significance of AIM2 upregulation, we investigated functions of AIM2 inflammasome in human monocyte‐macrophage lines. The AIM2 inflammasome agonist poly (dA:dT) works by binding to AIM2 molecules and causing AIM2 inflammasome assembly,^18^ whereas the inhibitor A151 works by competitively binding to AIM2 molecules and blocking AIM2 interaction with dsDNA thereby to hinder the assembly of inflammasome.^19^

**Figure 3:**
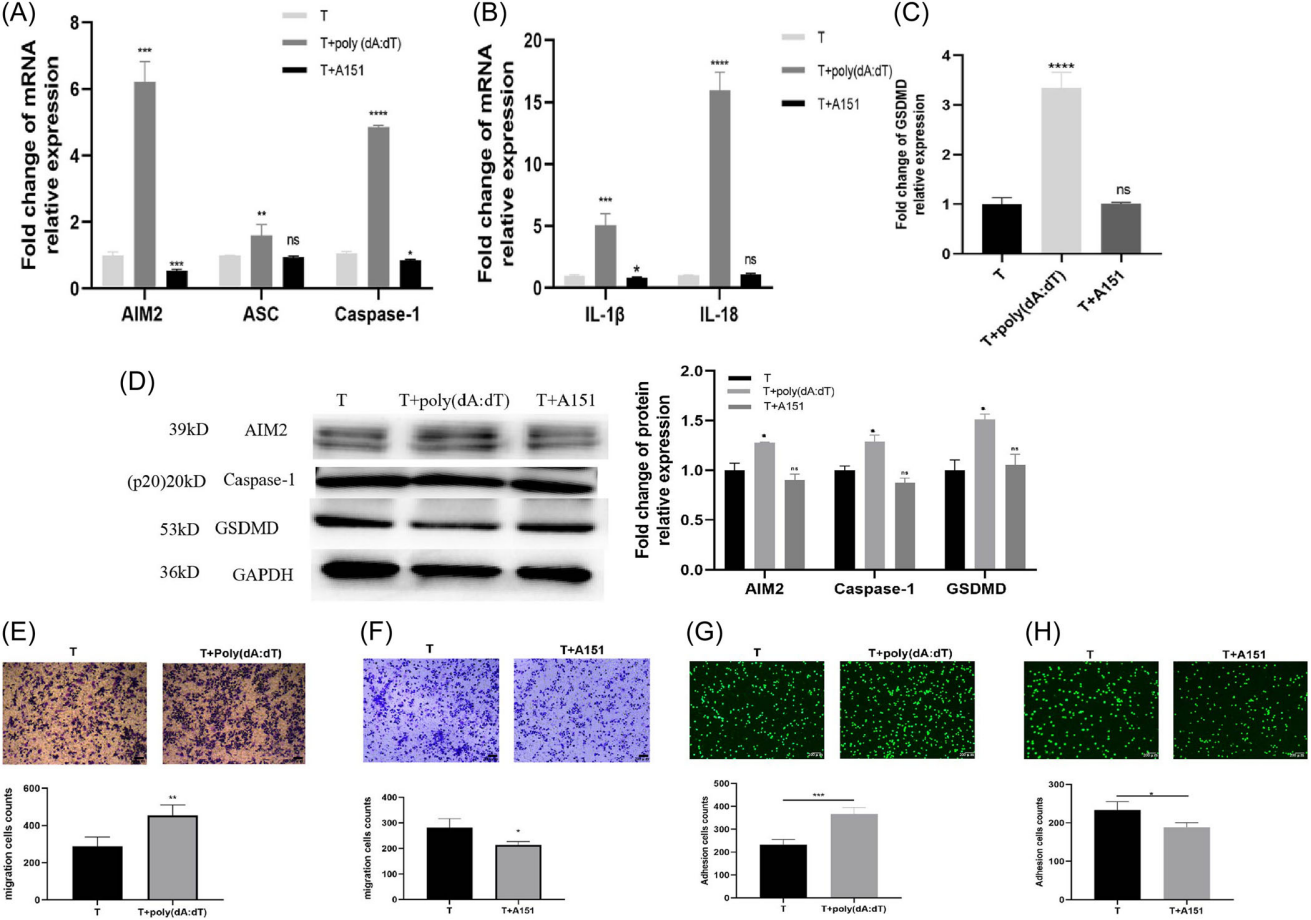
AIM2 inflammasome promotes inflammatory responses and causes pyroptosis in THP‐1 cells. The THP‐1 cells cultured in 0.2 μg/mL poly(dA:dT) with or without 3 μM A151 for 24 h. The mRNA and protein levels of AIM2, ASC, Caspase‐1, IL‐1β, IL‐18 and GSDMD were analyzed by qPCR (A−C) and Western blot analysis (D), respectively. The migration and adhesive capacity of THP‐1 cells was assessed using an inverted microscope (×100) (E, F) and an inverted fluorescence microscope (×100) (G, H), respectively. Scale bar, 100 μm. T, THP‐1 cells. Significant was defined as *p* < 0.05. *p < 0.05; **p < 0.01; ***p < 0.001; ****p < 0.0001. AIM2, Absent in Melanoma 2; CAD, Coronary Artery Disease; mRNA, messenger RNA; qPCR, quantitative PCR.

As shown in Figure 3A−D, expressions of AIM2, ASC and caspase‐1, and inflammatory factors (IL‐1β and IL‐18), and cell pyroptosis marker GSDMD were all increased, after the addition of poly(dA:dT). In contrast, the expressions of these genes decreased after the addition of A151. The same phenomenon was also observed in macrophages derived from THP‐1 monocytes, primary monocytes and their derived macrophages (Supporting Information S1: Figure 2). Compared with the control, the numbers of adhered and migrated THP‐1 cells were significantly increased after poly(dA:dT) treatments (Figure 3E,G). In contrast, A151 inhibited the adhesion and migration of THP‐1 cells (Figure 3F,H).

### 3.6 IFN‐γ promotes AIM2 inflammasome activation in THP‐1 cells

**Figure 4:**
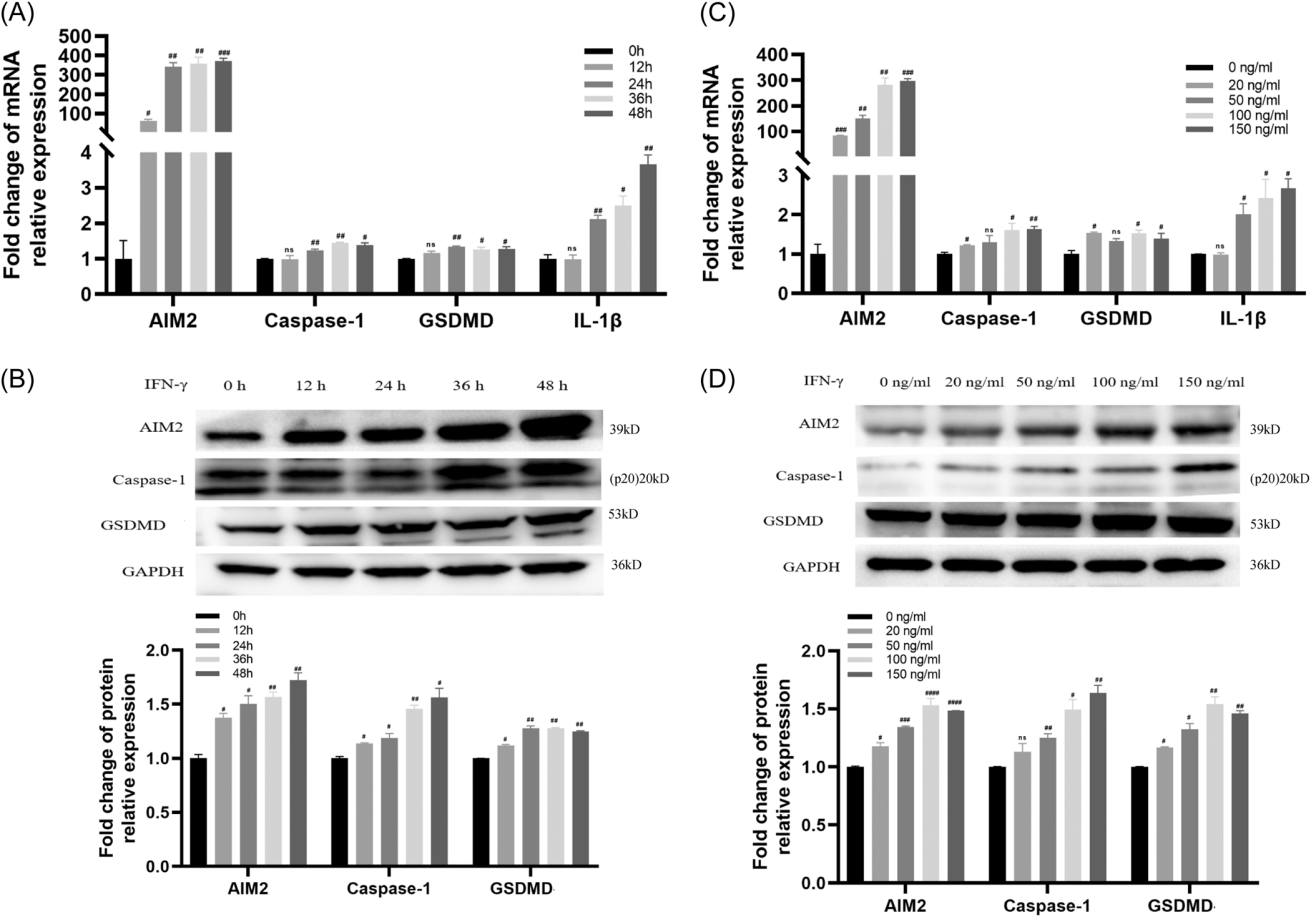
FN‐γ promotes the activation of AIM2 inflammasome and causes pyroptosis in THP‐1 cells. About 100 ng/mL IFN‐γ was used to culture THP‐1 cells for different time duration. The expression of AIM2, Caspase‐1, GSDMD, and IL‐1βwas analyzed using qPCR (A) and Western blot analysis (B). Different concentrations of IFN‐γ were used to culture THP‐1 cells for 24 h. The expression of AIM2, Caspase‐1, GSDMD, and IL‐1β was analyzed using qPCR (C) and Western blot analysis (D). Significant result was defined as *p* < 0.05. ^#^ *p* < 0.05; ^##^ *p* < 0.01; ^###^ *p* < 0.001; ^####^ *p* < 0.0001. AIM2, Absent in Melanoma 2; CAD, Coronary Artery Disease; mRNA, messenger RNA; qPCR, quantitative PCR.

Then, we explored the upstream signals that might induce AIM2 inflammasome activation in monocytes, which probably made AIM2 expressions an independent risk factor for CAD. Since IFN‐γ has been reported as an important precipitation factor in AS,^20^ and AIM2 is a member of the HIN200 family of IFN‐Stimulated Genes,^21^ we hypothesized IFN‐γ might be the upstream signal. Consistently, we found after IFN‐γ exposure, AIM2, AIM2 inflammasome components and its downstream genes were significantly upregulated (Figure 4A−D).

### 3.7 IFN‐γ‐induced activation of AIM2 inflammasome by the JAK2/STAT1 pathway

**Figure 5:**
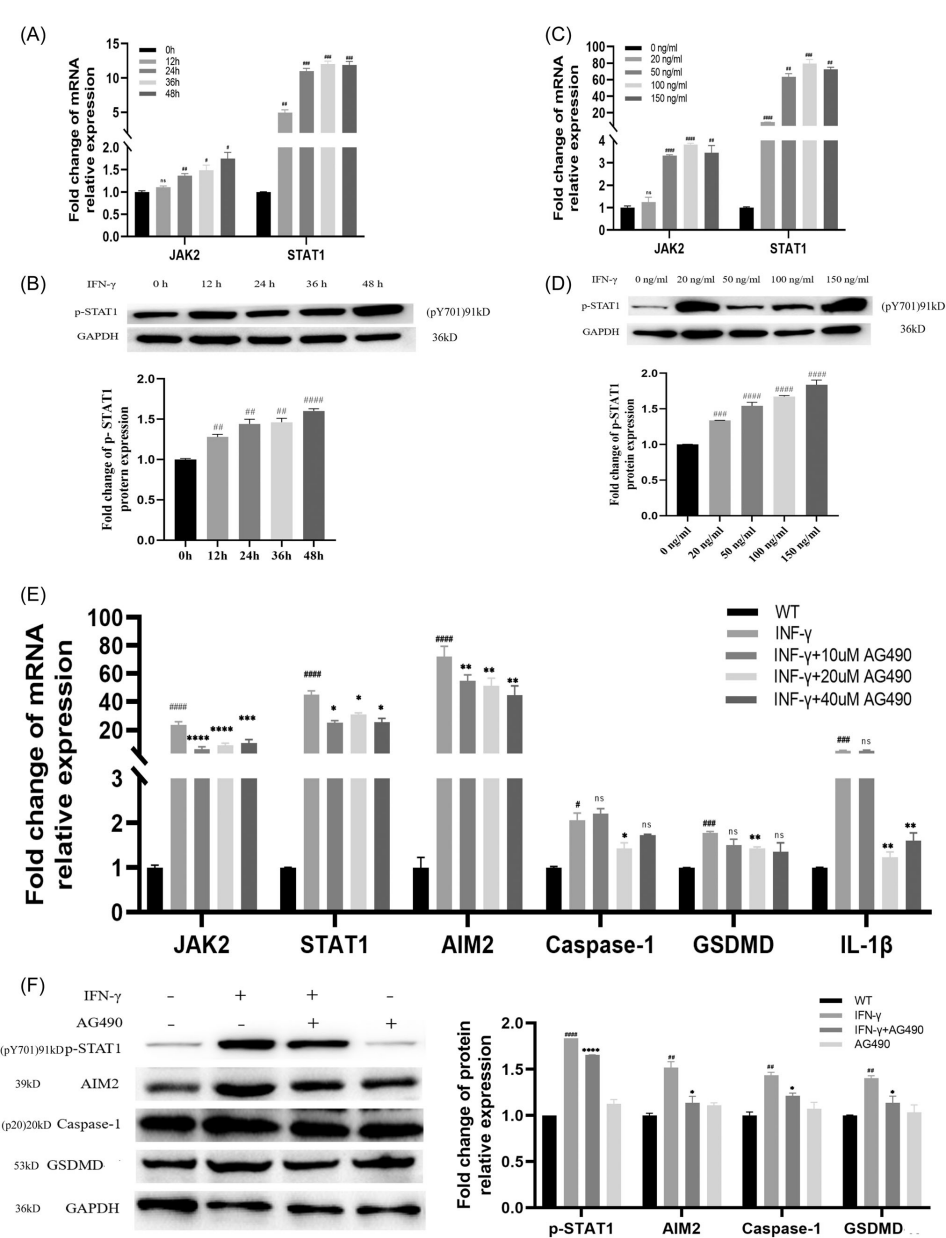
Pretreatment with AG490 prevents IFN‐γ‐induced activation of AIM2 inflammasome and pyroptosis in THP‐1 cells. THP‐1 cells were treated with 100 ng/mL IFN‐γ for different time duration, while the mRNA and protein levels of JAK2, STAT1 and p‐STAT1 were analyzed by qPCR (A) and Western blot analysis (B), respectively. THP‐1 cells were treated with variant concentrations of IFN‐γ for 24 h, while the mRNA and protein levels of JAK2, STAT1 and p‐STAT1 were analyzed by qPCR (C) and Western blot analysis (D), respectively. THP‐1 cells were pretreated with variant concentrations AG490 for 2 h, followed by treated with or without 100 ng/mL IFN‐γ for 24 h, while the mRNA (E) and protein levels (F) of key genes were analyzed by qPCR or Western blot analysis, respectively. Results are the means ± SDs for three independent experiments. Significant was defined as *p* < .05. Compared with the WT group, ^#^ *p* < 0.05; ^##^ *p* < 0.01; ^###^ *p* < 0.001; ^####^ *p* < 0.0001; Compared with the IFN‐γ‐treated group, *p < 0.05; **p < 0.01; ***p < 0.001; ****p < 0.0001. AIM2, Absent in Melanoma 2; CAD, Coronary Artery Disease; mRNA, messenger RNA; qPCR, quantitative PCR.

STAT1 protein is an important cytoplasmic transcription factor that can be induced by JAK2 phosphorylation in the presence of IFN‐γ.^22^ Consistent with others,^23^ we also found that IFN‐γ‐treated cells showed increased mRNA expressions of JAK2 and STAT1 (Figure 5A,C) and the protein expression of p‐STAT1 (Figure 5B,D). This suggests the activation of JAK2/STAT1 pathway.

JAK2 inhibitor AG490 was reported to achieve a blocking effect on the JAK2/STAT1 pathway,^8^, ^24^ so we pretreated THP‐1 cells with AG490 for 2 h, and then treated with the IFN‐γ for 24 h. The results showed that JAK2/STAT1 expressions were significantly reduced, as well as the phosphorylation of STAT1 protein, in the IFN‐γ + AG490 group. In addition, the addition of AG490 further reduced the expressions of AIM2 and its downstream molecules (Figure 5E,F). The results suggest that the IFN‐γ/JAK2/STAT1 pathway may be the upstream signaling molecules of AIM2 inflammasome.

### 3.8 AIM2 inhibitor A151/activator poly (dA:dT) attenuates/enhances adhesion and migration of THP‐1 cells after IFN‐γ treatment

**Figure 6:**
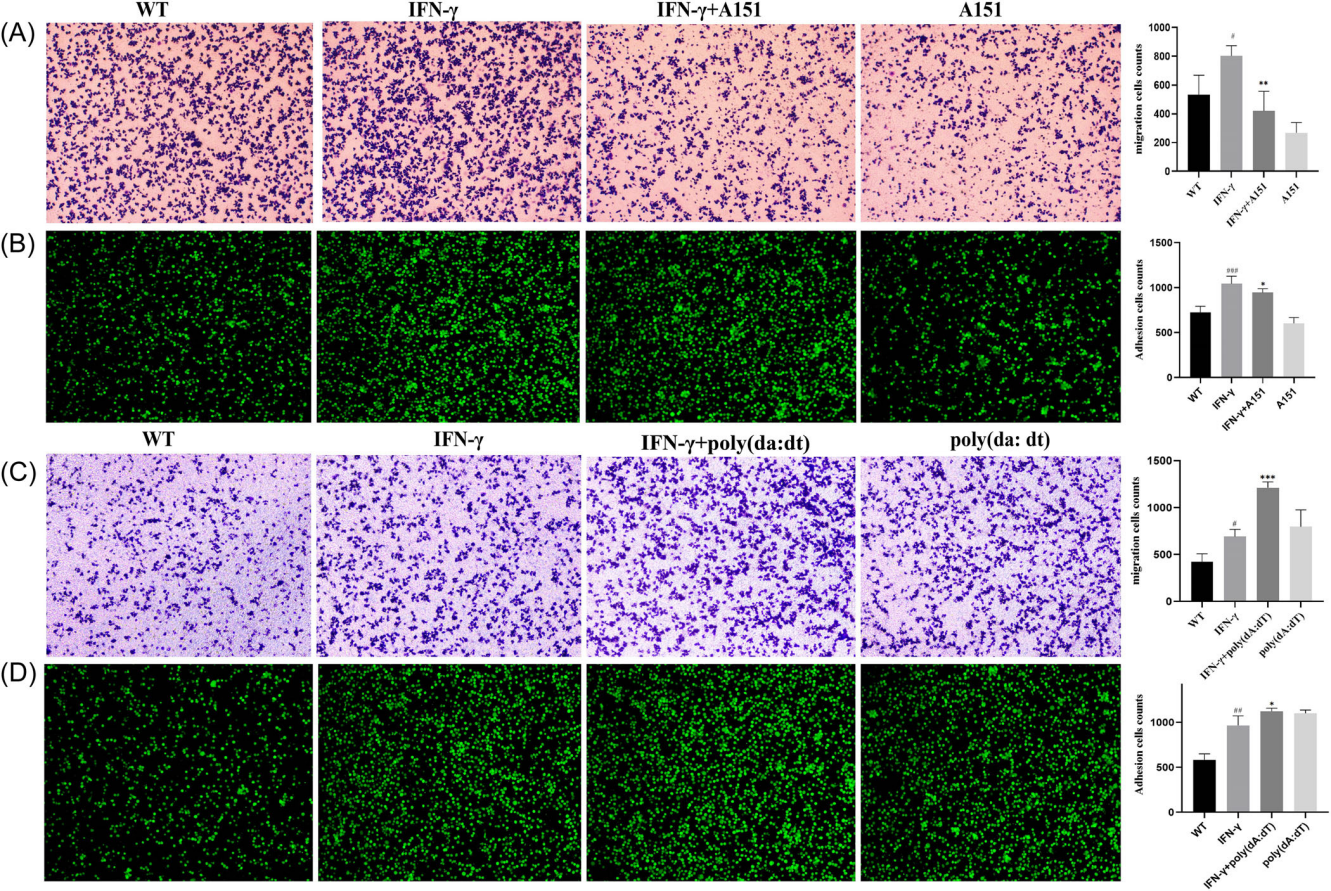
A151/poly(dA:dT) attenuates/enhances IFN‐γ‐induced adherent and migration of THP‐1 cells. THP‐1 cells were treated with 100 ng/mL IFN‐γ, followed by treated with 3 μM A151 or 0.2 μg/mL poly(dA:dT) for 24 h. The migration and adhesive capacity of THP‐1 cells was assessed using an inverted microscope (×100) (A, C) and an inverted fluorescence microscope (×100) (B, D), respectively. Scale bar, 100 μm. Significant was defined as *p* < 0.05. Compared with the WT group, ^#^ *p* < 0.05; ^###^ *p* < 0.001; Compared with the IFN‐γ‐treated group, *p < 0.05; **p < 0.01; ***p < 0.001. AIM2, Absent in Melanoma 2; CAD, Coronary Artery Disease; mRNA, messenger RNA; qPCR, quantitative PCR.

A151, an AIM2 inhibitor, attenuated the adhesion and migration of IFN‐γ‐treated THP‐1 cells, while the numbers of adhered and migrated THP‐1 cells were significantly increased after activator poly (dA:dT) treatment (Figure 6).

### 3.9 A151/poly(dA:dT) blocked/promoted IFN‐γ‐induced AIM2 inflammasome activation and pyroptosis

**Figure 7:**
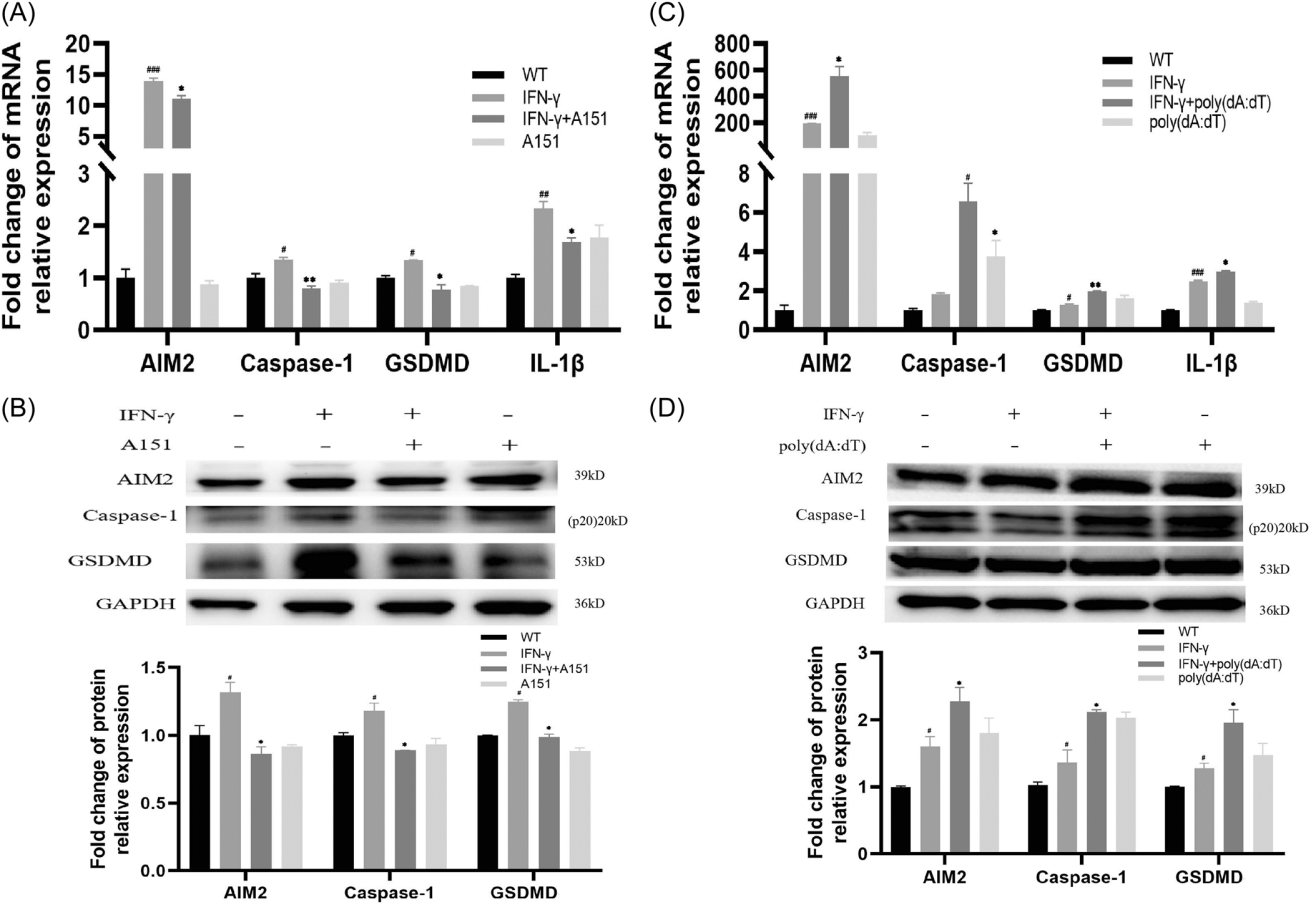
A151/poly(dA:dT) blocked/promoted IFN‐γ‐induced activation of AIM2 inflammasome and pyroptosis in THP‐1. THP‐1 cells were treated with 100 ng/mL IFN‐γ, followed by treated with 3 μM A151, 0.2 μg/mL poly(dA:dT) for 24 h. The mRNA (A, C and E) and protein (B, D) levels of AIM2, Caspase‐1, GSDMD and IL‐1β were presented, respectively. Significant was defined as *p* < 0.05. Compared with the WT group, ^#^ *p* < 0.05; ^##^ *p* < 0.01; ^###^ *p* < 0.001; Compared with the IFN‐γ‐treated group, *p < 0.05; **p < 0.01. AIM2, Absent in Melanoma 2; CAD, Coronary Artery Disease; mRNA, messenger RNA; qPCR, quantitative PCR.

Results showed that A151 effectively blocked the IFN‐γ‐induced activation of AIM2 inflammasome and alleviated pyroptosis, with the decrease of AIM2, Caspase‐1, GSDMD‐N and IL‐1β in THP‐1 cells (Figure 7A,B), while poly(dA:dT) promoted AIM2 inflammasome activation and pyroptosis in IFN‐γ pretreated THP‐1 cells (Figure 7C,D). Subsquently, we repeated our experiments with the inflammasome inhibitor “JC2‐11” and found that JC2‐11 effectively blocked IFN‐γ‐induced activation of AIM2 inflammasome in THP‐1 cells (Supporting Information S1: Figure 3), which further confirmed our results.

## 4 DISCUSSION

More and more experimental and clinical evidence suggest a chronic inflammation was participated in AS diseases, in which immune cells and various inflammatory factors play an important role.^25^, ^26^ Our study found that the high expression of AIM2 was an independent risk factor for CAD, that could promote inflammatory responses and pyroptosis in monocyte, which might be regulated by the IFN‐γ/JAK2/STAT1 pathway.^12^

Researchers first observed a significant increase of AIM2 in the necrotic core of AS plaques in 2014.^27^ Subsequently, it was reported that AIM2 could contribute to chronic inflammation by promoting the release of proinflammatory cytokines, and AIM2 expressions were correlated with plaque sizes.^28^ And the systemic inflammation in CAD could be evaluated by NLR, MLR, PLR and SII indices.^29^, ^30^, ^31^ Our results showed AIM2 was an independent risk factor for CAD, which is consistent with the finding by Pan et al.^28^ Our study also found that AIM2 was positively correlated with the neutrophil count, NLR, MLR, PLR, and SII, suggesting that AIM2 is possibly involved in the inflammatory process in CAD. The expressions of ASC and Caspase‐1 (AIM2 infalmmasome), GSDMD (the pyroptosis marker), and IL‐1β and IL‐18 (the proinflammatory factors) were all significantly upregulated in the CAD group, that further indicated the chronic inflammatory mechanism mediated by AIM2 inflammasome.

We treated THP‐1 monocytes, primary monocytes, and their derived macrophages with AIM2 agonist, then found a significant increase in AIM2 inflammasome components, cell pyroptosis marker, proinflammatory cytokines and the migration and adhesion ability of THP‐1 monocyte, while the opposite result was observed with AIM2 inhibitors A151 and inflammasome inhibitor JC2‐11.^32^

Pyroptosis is a pattern of programmed cell death accompanied by an inflammatory response, and it plays an important role in the inflammatory response associated with CAD.^33^, ^34^ We found that AIM2 overexpression could cause pyroptosis, and monocyte‐macrophage death in advanced AS lesions might promote the formation of necrotic nuclei and release inflammatory cytokines, chemokines, proteases, and other substances that greatly increase the risk of plaque rupture.^35^

Nevertheless, our study still has some limitations. First, the study population size is small, and some confounding factors may influence our results. Studies have shown that some other factors were involved in regulating of AIM2 expression. For example, Oxidized Low‐Density Lipoprotein (ox‐LDL) can increase the expression of AIM2 through the Nuclear Factor‐Κb signaling pathway.^28^ Primary cultured human aortic endothelial cells and human aortic smooth muscle cells show a rapid increase in endogenous AIM2 expression after exposure to TNF‐α or dsDNA stimulation.^27^, ^36^ Thus, other factors might also participant in regulating AIM2 expressions in monocytes, further study with multicenter large sample with extensive investigations on multiple confounding factors was required. Second, due to the less amount of monocytes in peripheral blood, our experiment was mainly performed in THP‐1 monocytes. Thirdly, we only used JAK2 inhibitor to treat the cells, while the experiment with JAK2 overexpression in THP‐1 cells could further verify the results.

## 5 CONCLUSIONS

In conclusion, we suggest that IFN‐γ may activate AIM2 inflammasome by the JAK2/STAT1 signaling pathway, triggering a series of subsequent inflammatory responses that promotes activation and pyroptosis in monocytes, and ultimately participate in the chronic inflammation in CAD.

## AUTHOR CONTRIBUTIONS

Bingyu Jin, Xiaokang Zhang and Yating Cheng collected the samples. Yue Zhao, Bin Liang, Shuyang Sheng and Chen Wang performed the experiments and analyzed the data. Fang Zheng and Changxin Shen wrote the manuscript. Fang Zheng conceived and designed the workflow and revised the manuscript. All authors read and approved the final manuscript.

## CONFLICT OF INTEREST STATEMENT

The authors declare no conflict of interest.

## Supporting information

Supporting information.

## Data Availability

All data generated or analyzed during this study are included in this article. GSE42148: (https://www.ncbi.nlm.nih.gov/geo/query/acc.cgi?acc=GSE42148).

## References

[1] Bahrami LS , Mohebaty M , Arabi SM , Tabesh H , Nematy M , Rezvani R . Effect of beetroot or beetroot plus vitamin C supplementation on cardiovascular function in patients with coronary artery disease: protocol for a double‐blind, placebo‐controlled, randomised trial. BMJ Open. 2022;12(6):e061394. 10.1136/bmjopen-2022-061394 PMC9204440 35710253

[2] Chan YH , Ramji DP . Key roles of inflammation in atherosclerosis: mediators involved in orchestrating the inflammatory response and its resolution in the disease along with therapeutic avenues targeting inflammation. Meth Mol Biol. 2022;2419:21‐37. 10.1007/978-1-0716-1924-7_2 35237956

[3] Ridker PM , Everett BM , Thuren T , et al. Antiinflammatory therapy with canakinumab for atherosclerotic disease. N Engl J Med. 2017;377(12):1119‐1131. 28845751 10.1056/NEJMoa1707914

[4] van Diepen JA , Thiem K , Stienstra R , Riksen NP , Tack CJ , Netea MG . Diabetes propels the risk for cardiovascular disease: sweet monocytes becoming aggressive? Cell Mol Life Sci. 2016;73(24):4675‐4684. 27469259 10.1007/s00018-016-2316-9 PMC5097107

[5] Poussin C , Laurent A , Peitsch MC , Hoeng J , De Leon H . Systems biology reveals cigarette Smoke‐Induced Concentration‐Dependent direct and indirect mechanisms that promote monocyte‐endothelial cell adhesion. Toxicol Sci. 2015;147(2):370‐385. 26141392 10.1093/toxsci/kfv137

[6] Razeghian‐Jahromi I , Karimi Akhormeh A , Razmkhah M , Zibaeenezhad MJ . Immune system and atherosclerosis: hostile or friendly relationship. Int J Immunopathol Pharmacol. 2022;36:039463202210921. 10.1177/03946320221092188 PMC9009140 35410514

[7] Ma Z , Wang C , Bai X , et al. TCF7 is highly expressed in immune cells on the atherosclerotic plaques, and regulating inflammatory signaling via NFκB/AKT/STAT1 signaling. Biosci Rep. 2022;42(7):BSR20212064. 35792753 10.1042/BSR20212064 PMC9297684

[8] Lee KM , Kang JH , Yun M , Lee SB . Quercetin inhibits the poly(dA:dT)‐induced secretion of IL‐18 via down‐regulation of the expressions of AIM2 and pro‐caspase‐1 by inhibiting the JAK2/STAT1 pathway in IFN‐γ‐primed human keratinocytes. Biochem Biophys Res Commun. 2018;503(1):116‐122. 29857000 10.1016/j.bbrc.2018.05.191

[9] Zhu H , Zhao M , Chang C , Chan V , Lu Q , Wu H . The complex role of AIM2 in autoimmune diseases and cancers. Immun Inflamm Dis. 2021;9(3):649‐665. 34014039 10.1002/iid3.443 PMC8342223

[10] Baatarjav C , Komada T , Karasawa T , et al. dsDNA‐induced AIM2 pyroptosis halts aberrant inflammation during rhabdomyolysis‐induced acute kidney injury. Cell Death Differ. 2022;29(12):2487‐2502. 35739254 10.1038/s41418-022-01033-9 PMC9750976

[11] Kim H , Seo JS , Lee SY , et al. AIM2 inflammasome contributes to brain injury and chronic post‐stroke cognitive impairment in mice. Brain Behav Immun. 2020;87:765‐776. 32201254 10.1016/j.bbi.2020.03.011

[12] Fidler TP , Xue C , Yalcinkaya M , et al. The AIM2 inflammasome exacerbates atherosclerosis in clonal haematopoiesis. Nature. 2021;592(7853):296‐301. 33731931 10.1038/s41586-021-03341-5 PMC8038646

[13] Lüsebrink E , Goody PR , Lahrmann C , et al. AIM2 stimulation impairs reendothelialization and promotes the development of atherosclerosis in mice. Front Cardiovasc Med. 2020;7:582482. 33263007 10.3389/fcvm.2020.582482 PMC7685997

[14] Pan J , Lu L , Wang X , et al. AIM2 regulates vascular smooth muscle cell migration in atherosclerosis. Biochem Biophys Res Commun. 2018;497(1):401‐409. 29448104 10.1016/j.bbrc.2018.02.094

[15] Rong J , Xu X , Xiang Y , et al. Thioredoxin‐interacting protein promotes activation and inflammation of monocytes with DNA demethylation in coronary artery disease. J Cell Mol Med. 2020;24(6):3560‐3571. 32039564 10.1111/jcmm.15045 PMC7131938

[16] Xiang Y , Liang B , Zhang X , et al. Atheroprotective mechanism by which folic acid regulates monocyte subsets and function through DNA methylation. Clin Epigenetics. 2022;14(1):32. 35227297 10.1186/s13148-022-01248-0 PMC8887029

[17] Lu L , Tong Y , Wang W , Hou Y , Dou H , Liu Z . Characterization and significance of monocytes in acute Stanford type B aortic dissection. J Immunol Res. 2020;2020:1‐15. 10.1155/2020/9670360 PMC7245667 32509885

[18] Li XQ , Yu Q , Fang B , Zhang ZL , Ma H . Knockdown of the AIM2 molecule attenuates ischemia‐reperfusion‐induced spinal neuronal pyroptosis by inhibiting AIM2 inflammasome activation and subsequent release of cleaved caspase‐1 and IL‐1β. Neuropharmacology. 2019;160:107661. 31181224 10.1016/j.neuropharm.2019.05.038

[19] Li Q , Cao Y , Dang C , et al. Inhibition of double‐strand DNA‐sensing cGAS ameliorates brain injury after ischemic stroke. EMBO Mol Med. 2020;12(4):e11002. 32239625 10.15252/emmm.201911002 PMC7136961

[20] Morana O , Nieto‐Garai JA , Björkholm P , et al. Identification of a new Cholesterol‐Binding site within the IFN‐γ receptor that is required for signal transduction. Adv Sci. 2022;9(11):e2105170. 10.1002/advs.202105170 PMC9008429 35166455

[21] Wang D , Zou J , Dai J , Cheng Z . Absent in melanoma 2 suppresses gastric cancer cell proliferation and migration via inactivation of AKT signaling pathway. Sci Rep. 2021;11(1):8235. 33859277 10.1038/s41598-021-87744-4 PMC8050218

[22] Liu Y , Ma X , Yang H , et al. APLNR regulates IFN‐γ signaling via β‐arrestin 1 mediated JAK‐STAT1 pathway in melanoma cells. Biochem J. 2022;479(3):385‐399. 35084016 10.1042/BCJ20210813

[23] Shao S , Tsoi LC , Sarkar MK , et al. IFN‐γ enhances cell‐mediated cytotoxicity against keratinocytes via JAK2/STAT1 in lichen planus. Sci Transl Med. 2019;11(511):eaav7561. 31554739 10.1126/scitranslmed.aav7561 PMC7285657

[24] Kobayashi A , Tanizaki Y , Kimura A , et al. AG490, a Jak2 inhibitor, suppressed the progression of murine ovarian cancer. Eur J Pharmacol. 2015;766:63‐75. 26410360 10.1016/j.ejphar.2015.09.039

[25] Hansen PR . Chronic inflammatory diseases and atherosclerotic cardiovascular disease: innocent bystanders or partners in crime? Curr Pharm Des. 2018;24(3):281‐290. 29318966 10.2174/1381612824666180110102341

[26] Ciurtin C , Robinson GA , Pineda‐Torra I , Jury EC . Challenges in implementing cardiovascular risk scores for assessment of young people with Childhood‐Onset autoimmune rheumatic conditions. Front Med. 2022;9:814905. 10.3389/fmed.2022.814905 PMC8883038 35237628

[27] Hakimi M , Peters A , Becker A , Böckler D , Dihlmann S . Inflammation‐related induction of absent in melanoma 2 (AIM2) in vascular cells and atherosclerotic lesions suggests a role in vascular pathogenesis. J Vasc Surg. 2014;59(3):794‐803.e2. 23790454 10.1016/j.jvs.2013.03.048

[28] Pan J , Han L , Guo J , et al. AIM2 accelerates the atherosclerotic plaque progressions in ApoE‐/‐ mice. Biochem Biophys Res Commun. 2018;498(3):487‐494. 29510138 10.1016/j.bbrc.2018.03.005

[29] Akin F , Ayca B , Celik O , Sahin C . Predictors of poor coronary collateral development in patients with stable coronary artery disease: neutrophil‐to‐lymphocyte ratio and platelets. Anadolu Kardiyoloji Dergisi Anatolian J Cardiol. 2015;15(3):218‐223. 10.5152/akd.2014.5263 PMC5337058 25880175

[30] Balkarli A , Kucuk A , Babur H , Erbasan F . Neutrophil/lymphocyte ratio and mean platelet volume in Behçet's disease. Eur Rev Med Pharmacol Sci. 2016;20(14):3045‐3050. 27460734

[31] Montecucco F , Liberale L , Bonaventura A , Vecchiè A , Dallegri F , Carbone F . The role of inflammation in cardiovascular outcome. Curr Atheroscler Rep. 2017;19(3):11. 28194569 10.1007/s11883-017-0646-1

[32] Lee G , Ahn H , Yun J‐H , et al. JC2‐11, a benzylideneacetophenone derivative, attenuates inflammasome activation. Sci Rep. 2022;12(1):22484. 36577816 10.1038/s41598-022-27129-3 PMC9797494

[33] Song D , Li M , Yu X , et al. The molecular pathways of pyroptosis in atherosclerosis. Front Cell Dev Biol. 2022;10:824165. 35237603 10.3389/fcell.2022.824165 PMC8884404

[34] Chen X , Tian PC , Wang K , Wang M , Wang K . Pyroptosis: role and mechanisms in cardiovascular disease. Front Cardiovasc Med. 2022;9:897815. 35647057 10.3389/fcvm.2022.897815 PMC9130572

[35] Gerasimova EV , Popkova TV , Gerasimova DA , Kirichenko TV . Macrophage dysfunction in autoimmune rheumatic diseases and atherosclerosis. Int J Mol Sci. 2022;23(9):4513. 35562903 10.3390/ijms23094513 PMC9102949

[36] Wortmann M , Skorubskaya E , Peters AS , Hakimi M , Böckler D , Dihlmann S . Necrotic cell debris induces a NF‐κB‐driven inflammasome response in vascular smooth muscle cells derived from abdominal aortic aneurysms (AAA‐SMC). Biochem Biophys Res Commun. 2019;511(2):343‐349. 30782482 10.1016/j.bbrc.2019.02.051

