# Supplementary material for: AIM2 inflammasome regulated by the IFN‐γ/JAK2/STAT1 pathway promotes activation and pyroptosis of monocytes in Coronary Artery Disease
Source: Immun Inflamm Dis. 2024 Jun 13;12(6):e1317. doi: 10.1002/iid3.1317 (PMC11170685; doi:10.1002/iid3.1317)
Supplement: Supplementary file 1 — Supporting information. [file IID3-12-e1317-s001.docx]

| **Supplementary Table 1** Primer sequences for Real-time PCR | | | | |
| --- | --- | --- | --- | --- |
| Gene | species | Forward (5' → 3') | | Reverse (5' → 3') |
| AIM2 | human | TGGCAAAACGTCTTCAGGAGG | | AGCTTGACTTAGTGGCTTTGG |
| ASC | human | AGCTCACCGCTAACGTGCTGC | | GCTTGGCTGCCGACTGAGGAG |
| Caspase-1 | human | | GCTGAGGTTGACATCACAGGCA | TGCTGTCAGAGGTCTTGTGCTC |
| GSDMD | human | | GGACCCTAACACCTGGCAGACT | TTGTGGGTGCGCGTGACTT |
| IL-1β | human | | ATGATGGCTTATTACAGTGGCAA | GTCGGAGATTCGTAGCTGGA |
| IL-18 | human | | GCTGAAGATGATGAAAACCTGGA | GAGGCCGATTTCCTTGGTCA |
| JAK2 | human | | AGCCTATCGGCATGGAATATCT | TAACACTGCCATCCCAAGACA |
| STAT1 | human | | ATGGCAGTCTGGCGGCTGAATT | CCAAACCAGGCTGGCACAATTG |
| GAPDH | human | | TGTTGCCATCAATGACCCCTT | CTCCACGACGTACTCAGCG |

GSE42148 11 CON vs 11 CAD


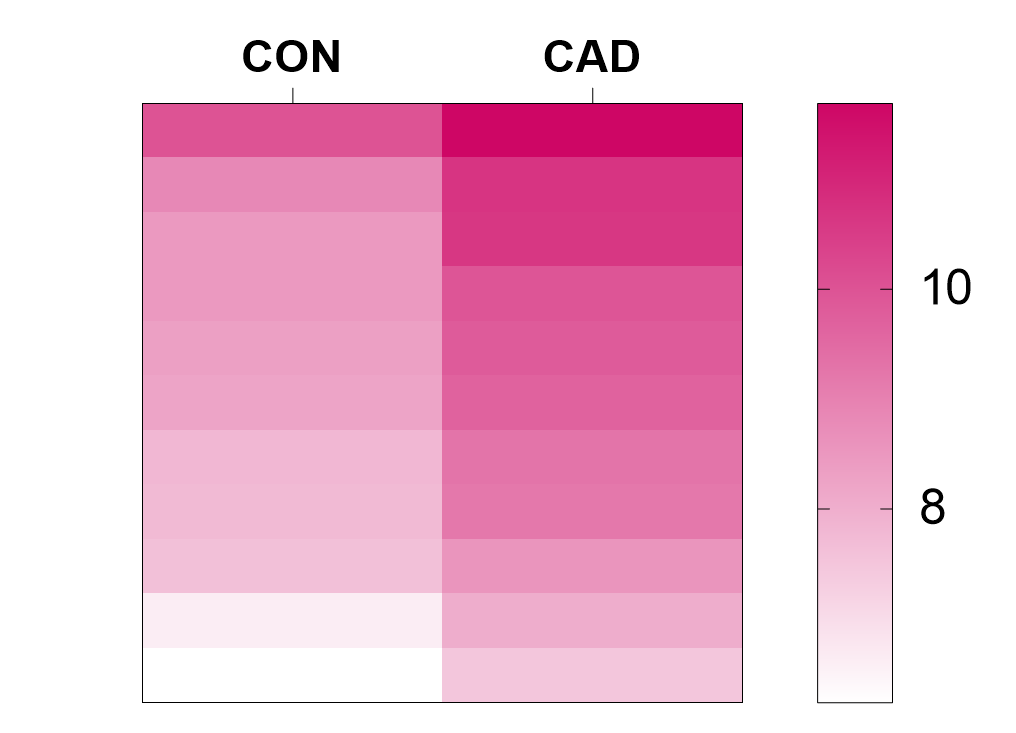


**Supplementary Figure 1** Differential expression of the AIM2 gene was present in the control and CAD groups in the GSE42148 microarray.


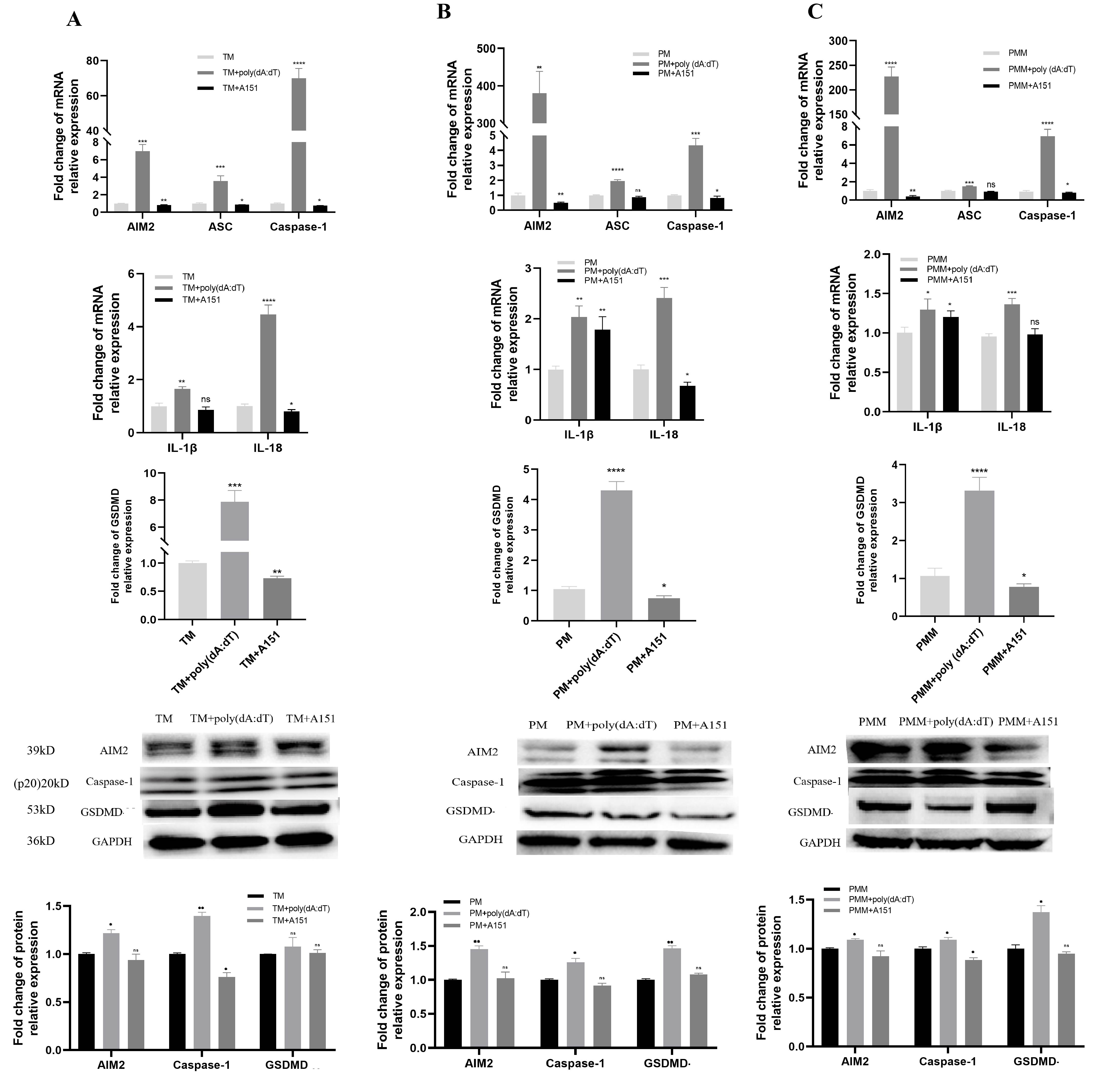


**Supplementary Figure 2** AIM2 inflammasome promotes inflammatory responses and causes pyroptosis in human monocyte lines. Human monocytes were treated with 0.2 μg/ml poly(dA:dT) or 3μM A151 for 24h. The mRNA and protein levels of AIM2, ASC, Caspase-1, IL-1β, IL-18 and GSDMD in macrophages derived from THP-1 monocytes (A), primary monocytes (B) and their derived macrophages (C) were analyzed by qPCR and western blotting, respectively. TM, THP-1 derived Macrophages; PM, Primary Monocytes PMM, PM derived Macrophages. *p* < 0.05 was defined as significant. ^*^ *p* < 0.05; ^**^ *p* < 0.01; ^***^ *p* < 0.001; ^****^ *p* < 0.0001


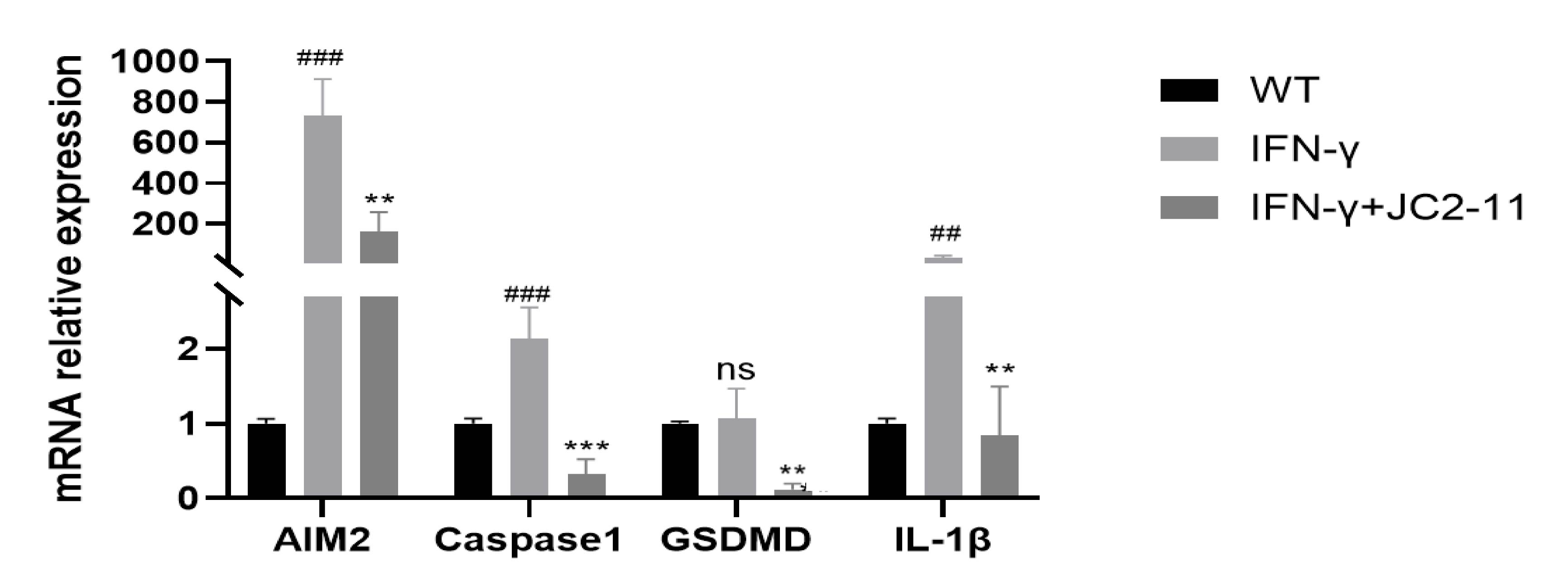


**Supplementary Figure 3** JC2-11 blocked IFN-γ-induced activation of AIM2 inflammasome in THP-1. THP-1 cells were treated with 100 ng/ml IFN-γ, followed by treated with 50μM JC2-11 for 24h. The mRNA levels of AIM2, Caspase1, GSDMD and IL-1β were presented, respectively. Significant was defined as *p* < 0.05. Compared with the WT group, ^##^*p* < 0.01; ^###^*p* < 0.001; Compared with the IFN-γ-treated group, ***p* < 0.05; ****p* < 0.01.
